# Accurate modeling of the potential energy surface of atmospheric molecular clusters boosted by neural networks†

**DOI:** 10.1039/d4va00255e

**Published:** 2024-08-13

**Authors:** Jakub Kubečka, Daniel Ayoubi, Zeyuan Tang, Yosef Knattrup, Morten Engsvang, Haide Wu, Jonas Elm

**Affiliations:** a Department of Chemistry, Aarhus University Langelandsgade 140 8000 Aarhus C Denmark ja-kub-ecka@chem.au.dk +420 724946622; b Center for Interstellar Catalysis, Department of Physics and Astronomy, Aarhus University Ny Munkegade 120 8000 Aarhus C Denmark

## Abstract

The computational cost of accurate quantum chemistry (QC) calculations of large molecular systems can often be unbearably high. Machine learning offers a lower computational cost compared to QC methods while maintaining their accuracy. In this study, we employ the polarizable atom interaction neural network (PaiNN) architecture to train and model the potential energy surface of molecular clusters relevant to atmospheric new particle formation, such as sulfuric acid–ammonia clusters. We compare the differences between PaiNN and previous kernel ridge regression modeling for the Clusteromics I–V data sets. We showcase three models capable of predicting electronic binding energies and interatomic forces with mean absolute errors of <0.3 kcal mol^−1^ and <0.2 kcal mol^−1^ Å^−1^, respectively. Furthermore, we demonstrate that the error of the modeled properties remains below the chemical accuracy of 1 kcal mol^−1^ even for clusters vastly larger than those in the training database (up to (H_2_SO_4_)_15_(NH_3_)_15_ clusters, containing 30 molecules). Consequently, we emphasize the potential applications of these models for faster and more thorough configurational sampling and for boosting molecular dynamics studies of large atmospheric molecular clusters.

## Abstract 2

We present the application of machine learning methods to alleviate the computational cost of quantum chemistry calculations required for modeling atmospheric molecular clusters.

## Graphical abstract

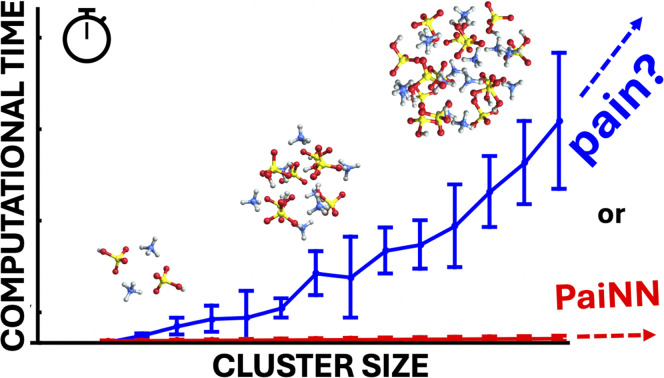

**Environmental significance**

Atmospheric aerosol particles significantly impact human health, atmospheric chemistry, and climate. Yet, aerosol processes remain poorly understood, introducing major uncertainties in climate models. New particle formation is a process driven by formation of stable molecular clusters that grow into aerosols. While theoretical and experimental studies qualitatively agree for simple systems, massive errors are observed in complex systems. Unfortunately, accurate quantum chemical calculations for these systems are computationally demanding. However, machine learning can replicate those at a fraction of the computational cost. We trained neural networks on various systems and demonstrated their effectiveness for large molecular clusters. This is a significant step toward large-scale, *ab initio* modeling of atmospheric nucleation that will potentially reduce uncertainties in climate predictions.

## 1 Introduction

The formation and growth of molecular clusters in the atmosphere drive the gas-to-particle conversion process known as new particle formation (NPF). These aerosols contribute to a net cooling effect on the Earth.^1^ Almost 50% of newly formed aerosols act as cloud condensation nuclei (CCN), enhancing cloud formation and the albedo effect.^2,3^ Additionally, due to their tiny size, aerosols can transport various molecules, viruses, and bacteria deeply into the respiratory system, posing health risks.^4–6^

While the exact chemical species relevant to aerosol formation mechanisms are poorly understood, many studies present insight into the most relevant components, such as sulfuric acid, ammonia, dimethylamine, and various oxidation products of volatile organic compounds.^7–10^ Other studies have employed computational quantum chemistry (QC) and modeling to investigate the first crucial steps of formation mechanisms; molecular cluster formation.^11–13^ One of the main paths is through stable inorganic acid–base salts. Accurate QC methods are required to capture the chemistry of common acid–base clusters, which are stabilized by proton transfer; a bond-breaking/-formation reaction. However, these methods are computationally expensive and scale significantly with molecular size, which often limits computational studies to small clusters, typically with less than 10 molecules. To overcome the computational limitation while maintaining accuracy, alternative approaches for future practical research are needed.

Machine learning (ML) offers a versatile and powerful solution for accelerating time-consuming processes characterized by repeating patterns.^14^ In chemistry, ML has found applications in modeling potential energy surfaces (PES), predicting molecular structures, identifying and classifying molecules, enhancing molecular dynamics (MD) simulations, predicting kinetics and other properties, and gaining deeper insights into molecular behavior.^15–20^ To date, and to the best of our knowledge, there are few studies utilizing ML for atmospheric molecular clusters.^21–25^ Our recent studies focused on using kernel ridge regression (KRR) models (implemented in QML^26,27^) for modeling the cluster binding energies.^28–33^ Despite its simplicity, KRR achieved chemical accuracy (<1 kcal mol^−1^) when modeling the energies of sulfuric acid–water clusters, using only a few hundred structures for training and the rest of the database for testing.^28^ However, Knattrup *et al.*^34^ demonstrated that the computational costs and accuracy of KRR for modeling of density functional theory (DFT) binding energies could be substituted by fast but (compared to DFT) less accurate DFT-3c^35,36^ methods with similar results. Moreover, for accurate modeling, the computational costs of the KRR approach (kernel construction and Cholesky decomposition) almost reach those of QC methods. Additionally, enlarging the configurational space by incorporating more non-equilibrium structures and increasing system complexity by introducing more atom/molecule types would require very large training databases to maintain low errors, increasing costs even further.

This work investigates whether neural networks can address these challenges. We utilize one of the commonly applied NN architectures, the polarizable atom interaction neural network (PaiNN),^37^ implemented in SchNetPack,^38,39^ to model the energies and forces of typical atmospheric molecular clusters. We demonstrate the accuracy and speed of the trained models and discuss their potential applications in future studies, including those involving large and complex training databases.

## 2 Methodology

### 2.1 Databases

**Fig. 1:**
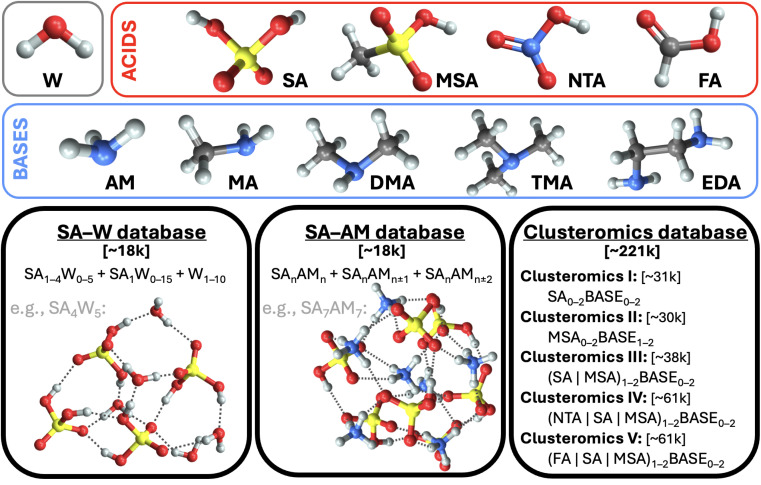
The three databases of molecular cluster structures used in this work. Legend: water (W), ammonia (AM), methylamine (MA), dimethylamine (DMA), trimethylamine (TMA), ethylenediamine (EDA), and sulfuric (SA), methanesulfonic (MSA), nitric (NTA), and formic (FA) acids. Color code: sulfur (yellow), oxygen (red), hydrogen (white), nitrogen (blue), carbon (gray).

This work uses structures from three molecular cluster databases (*cf.*Fig. 1 and SI-1†). The first is the sulfuric acid (SA) and water (W) system studied in our previous work.^28^ To sum up, we had collected ∼1.7k equilibrium structures from other studies^40–48^ and used Born–Oppenheimer MD (BOMD) simulations with energies and forces computed at a low QC level (the PM7 semi-empirical method) starting from each equilibrium structure in order to expand the database with several non-equilibrium structures. Thus, overall, this database contains ∼18k structures, which consist of a subset of the SA_0–5_W_0–15_ clusters. The system properties are evaluated for each geometry at the ωB97X-D^49^/6-31++G(d,p) level of theory.

The second SA and ammonia (AM) cluster database has been taken from Engsvang and Elm.^29^ They used the GFN1-xTB^50,51^ level of theory to produce ∼4k equilibrium structures and ∼7.7k non-equilibrium structures. In this work, we used the equilibrium structures as starting points for BOMD simulations at 300 K while producing an additional ∼6.4k structures. The overall database thus consists of ∼18k SA_*n*_AM_*n*,*n*±1,*n*±2_ clusters, where *n* ∈ 1–9. The GFN1-xTB level cannot provide accurate binding energies, and using ωB97X-D/6-31++G(d,p) would be too computationally demanding for all cluster sizes. Therefore, using the same methodology as Engsvang and Elm,^29^ the energies and forces were recalculated at the B97-3c^35^ level.

Although both databases are similar in size, the SA–AM database contains larger clusters, an extra atom type (nitrogen), and a more complex scheme of cluster binding patterns than just O–H interactions, as in the SA–W database. We additionally recalculated the SA–W database at the B97-3c^35^ level. Therefore, we have three different datasets (SA–W^ωB97X-D/6-31++G(d,p)^, SA–W^B97-3c^, and SA–AM^B97-3c^) to compare for the suitability of ML. As such, we can examine the role of the theory level and database complexity in ML training.

Finally, the third database of structures originates from Knattrup *et al.*,^32^ who compiled small (up to 4-molecule) clusters composed of various NPF precursors such as (SA, MSA = methanesulfonic, NTA = nitric, and FA = formic) acids and (AM, MA = methylamine, DMA = dimethylamine, TMA = trimethylamine, and EDA = ethylenediamine) bases. Knattrup *et al.* combined the Clusteromics I–V databases^12,52–55^ consisting of equilibrium structures optimized at ωB97X-D/6-31++G(d,p). Furthermore, they employed MD simulations at GFN1-xTB^50,51^ to greatly expand the database to ∼221k structures, denoted Clusterome. Ultimately, the system properties (single-point energies) were evaluated for each geometry at r^2^SCAN-3c.^36^ The entire Clusterome database offers an opportunity to examine the capability of NN for learning on structurally complex (clusters composed of nine different molecules) and large databases or appropriately reduced databases.^56^

We emphasize the importance of consistency during data preparation. For instance, a mismatch of quantum chemistry (QC) program versions or methods could cause great errors in the modeling. Therefore, to deal with the database and the QC evaluations systematically, we have used the JK framework, a set of computational tools for handling molecular clusters.^33^ The following QC programs were used for QC calculations: xtb 6.4,^57^ Gaussian 16 B.01,^58^ and ORCA 5.0.3.^59,60^

### 2.2 Data properties

Each cluster structure (*XYZ* Cartesian coordinates) in the databases has an associated electronic energy and, in some cases, also forces derived from electronic energy gradients. For ML modeling, the structure must have a suitable molecular representation (explained in Section 2.3) since different *XYZ* coordinates can correspond to the same structure when translated, mirrored, or atom-wise permuted since *XYZ*s are translation/rotation/atom-permutation noninvariant. Furthermore, relative energies (*e.g.*, atomization or binding energies) are preferable to absolute electronic energies, as they exhibit a lower spread of the modeled values between different clusters, which simplifies the data fitting. Therefore, we use the electronic binding energies Δ*E*, *i.e.*, the energies released upon cluster (C) formation from its monomers (M) at energy-minimum configuration1
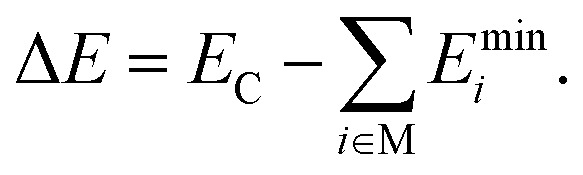
The gradient of *E*_C_ is the same as for Δ*E*, *i.e.*, still corresponding to intramolecular forces. When evaluating the ML model quality, we use mean absolute errors (MAEs) and root mean squared errors (RMSEs) between the predicted and true properties. In the case of interatomic forces 
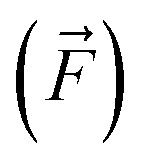
, we compare all force components separately.

In our previous work,^28^ we demonstrated that the Δ-ML approach^61–65^ could enhance the accuracy of predictions by a factor of four compared to direct-ML. Here, direct learning refers to modeling the high-level binding energy (here denoted as Δ*E*^high^). Δ-ML models the difference (ΔΔ*E*) between a slow, high-level theory (Δ*E*^high^) and a fast, low-level theory (*e.g.*, GFN1-xTB,^50,51^ here denoted as Δ*E*^low^),

2 ΔΔ*E* = Δ*E*^high^ − Δ*E*^low^.

If the low- and high-level methods correlate, the model does not need to learn as much chemistry, as the fast method captures most of it. While the combination of Δ-ML and NN is uncommon as the improvement is not marginal and Δ-ML is less practical for final application (*e.g.*, MD simulations), we only use it for consistency and eventual comparisons with our previous studies. Therefore, when Δ-ML is used, we used GFN1-xTB as the ‘low’ method.

### 2.3 Neural network model

In this work, we use the polarizable atom interaction NN (PaiNN^37^) model, the successor to the SchNet^66^ model. Based on Liao *et al.*,^67^ other NN models (*e.g.*, TorchMD-NET,^68^ NequIP,^69^ and Equiformer^67^) can achieve better accuracy when modeling energies and forces. However, these models require longer training times while the accuracy (MAEs) is only improved by a factor of two or less.

PaiNN employs message passing for 3D-embedded graphs, where the molecular representation is constructed on the fly for each graph node (atom) based on its surrounding environment (by default 5 Å). Atomic features are updated through a sequence of interaction layers, which, followed by an atom-centered neural network, allows modeling of the final property of interest (*e.g.*, energy). For a more detailed description, we recommend consulting the original reference.^37^

The PaiNN model is implemented in the SchNetPack^38,39^ program. Communication with SchNetPack is also performed *via* the JK framework.^33^

#### 2.3.1 Model hyperparameters

Hyperparameters investigated in this work include molecular representation, neural network, and training settings that can significantly impact the model accuracy and performance. For PaiNN, some crucial hyperparameters are the initial learning rate (LR), number of epochs (EPOCHS; training length), batch size (BS), number of interaction layers (INT), number of atom basis features (AB), and number of radial basis functions (RB) within a cutoff distance (CUTOFF).

#### 2.3.2 Model training

The quality of training is determined by MAE between the modeled and true values. Typically, the majority (we use 90%) of the training database is used as the training subset, and through a sequence of batch iterations/epochs, the MAE is minimized. The remaining portion (10%) of the database is used for validation to prevent overfitting of the training database. In such cases, the training is often stopped when the validation MAE consistently increases. However, there are two other cases when the training is typically stopped: (1) when the validation MAE has reached a plateau, *i.e.*, no improvement occurred for a certain number of epochs (early stopping, ES, threshold), or (2) the validation MAE has reached the desired accuracy.

When training a model with multiple properties, it is common to use a loss function combining the MAEs of all the model properties. In the case of energies and forces, our loss function is3
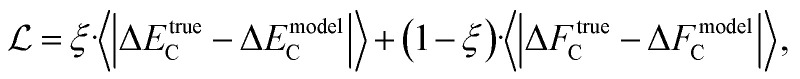
where 〈|·|〉 corresponds to MAE and *ξ* to trade-off between the two modeled properties. This work sets the trade-off to 1% when training on both energies and forces.

Training on the full database can become computationally demanding in the case of very large databases. Smith *et al.*^56^ suggested selecting a small subset of the full database (*e.g.*, 2%) and training several models, each initiated with a different random seed. The largest deviations (or deviations greater than a certain cutoff) between the models' predictions on the remaining part (98%) of the database can be used to identify and select the problematic structures for expanding the training database. This process is iteratively repeated until the final trained models consistently predict properties of the full database within the desired accuracy. We test this database reduction in Section 3.5.

## 3 Results

### 3.1 Understanding the NN training

#### 3.1.1 Training curves

We will first investigate the training behavior before moving towards the NN-model training on the full databases. Here, we used two NN models (NN-small and NN-big) defined by choosing suitable hyperparameters based on intuition. The NN-small model (BS = 100, LR = 10^−4^, AB = 64, INT = 3, RB = 15, and CUTOFF = 5 Å) contained 154k trainable parameters and NN-big (BS = 100, LR = 10^−4^, AB = 256, INT = 5, RB = 30, and CUTOFF = 5 Å) contained 3.8 M trainable parameters. The other hyperparameters were the same as for the final model (see section 3.1.2). The models have been separately trained on electronic binding energies (Δ*E*) for random samples of 1k and 16k structures from the SA–W database.

**Fig. 2:**
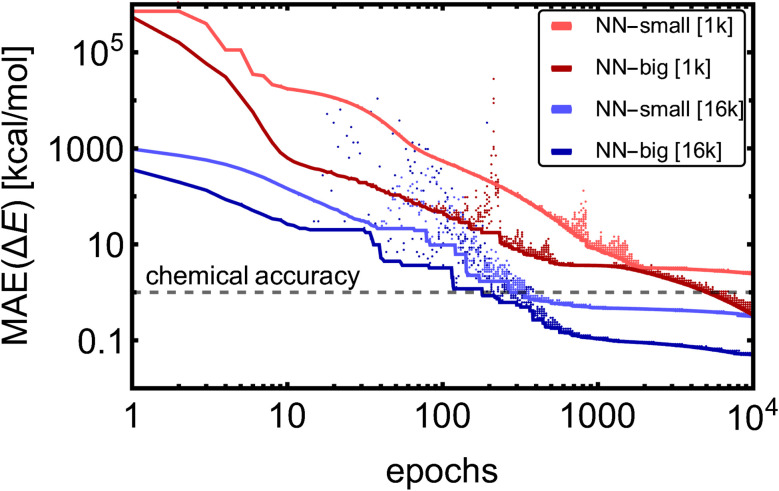
Mean absolute errors (MAEs) of electronic binding energies (Δ*E*) for the training SA–W database with different training set sizes (1k/16k) and different numbers of NN fitting parameters (NN-small/NN-big). Dots show the training MAE from each epoch, and lines show the MAE of the best model found so far.

Fig. 2 shows the training MAE evolutions for the four training cases. Due to the model complexity, the NN-big models reached training MAEs of 0.05 kcal mol^−1^ [16k] and 0.3 kcal mol^−1^ [1k], an order of magnitude lower MAEs than their NN-small counterparts, which reached MAEs of 0.3 kcal mol^−1^ [16k] and 2.5 kcal mol^−1^ [1k]. After a few hundred to several thousand epochs, only fine-tuning of the accuracy occurs, except for the case of NN-big [1k], where the fitting accuracy is still significantly improved. It should be noted that in the case of NN-big [1k], the final improvement might already be overfitting the training database, and validation should be examined simultaneously (see Section 3.1.3). Yet, these results do not show a clear trend between the NN model complexity and the number of epochs required to reach the fine-tuning regime. The larger training datasets [16k] converge approximately 16 times faster to similar values compared to the small datasets [1k] because we use 16 times more training batches within each epoch. To sum up, the training MAEs of NN-small [1k] can be lowered by ∼2 orders magnitude by greater variation in the training datasets [1k → 16k] and by enlarging the model [NN-small → NN-big].

Training a moderately complex neural network from scratch on a large dataset boosted by a GPU takes from several hours to a few days. However, this can vary widely depending on the specific circumstances: model complexity in the form of the number of fittable parameters, training dataset size and complexity (*e.g.*, 1k/16k data of SA–W/SA–AM will differ), hardware (number and speed of CPUs/GPUs), optimization technique (*e.g.*, Adam or stochastic gradient descent (SGD^70^)), learning strategy (*e.g.*, learning rate or transfer learning = adaptive learning), and number of epochs. Training is the bottleneck of NN modeling, as predicting properties of thousands of structures takes a couple of minutes when employing a single CPU. Table 1 illustrates the training times required for one epoch of different datasets. Various commonly-used hyperparameters can alter the computational time by a factor of 0.5–2. The main conclusions from Table 1 are that the training on larger databases (16k *vs.* 1k) is proportionally slower (*i.e.*, 16 times), training on more complex databases (SA–AM *vs.* SA–W) is slightly slower (by ∼25%), and training more properties (
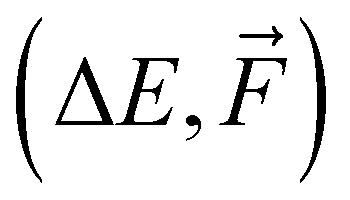
*vs.* Δ*E*) doubles the times in our case. While utilizing GPUs make the training faster by more than an order of magnitude, we typically only have access to a few GPUs but can utilize thousands of CPUs. Therefore, the wide range of training runs required for hyperparameter optimization is performed using only CPUs with fewer epochs.

**Table 1:** Approximate computational times for training cases and hardware setups. Here, *x* CPU = *x* Intel Xeon Platinum cores, and 1 GPU + 4 CPUs = Nvidia V100-16GB + 4 Intel Xeon Gold cores

System	Training on	Train size	1 CPU	4 CPUs	1 GPU + 4 CPUs
SA–W	Δ*E*	1k	∼4 min per epoch	∼2 min per epoch	∼8 s per epoch
SA–W	Δ*E*	16k	∼1 h per epoch	∼0.5 h per epoch	∼50 s per epoch
SA–W	Δ*E* + forces	1k	∼8 min per epoch	∼4 min per epoch	∼16 s per epoch
SA–AM	Δ*E*	1k	∼6 min per epoch	∼3 min per epoch	∼10 s per epoch

#### 3.1.2 Hyperparameter optimization

Non-surprisingly, the previous section illustrates that model complexity and training set size have an impact on the accuracy of the final model. Using the same training database, it is clear after a few (10–100 s) epochs which set of hyperparameters performs better in training (see Fig. 2). Such an assumption is not universally valid as some setups could converge slower but to more accurate values. Nevertheless, with this approach, we should be able to find the model that most rapidly converges to low MAEs. Therefore, in this section, we seek hyperparameters that reach the lowest validation MAEs after 200 epochs.

Taking into account the most important hyperparameters (AB, INT, RB, LR, and BS), we used an in-house numerical optimization script to find optimal hyperparameters for the NN-model trained on Δ*E* of 1k SA–W data. However, as the outcome of NN training is not predictable and depends on the initial seeding of the network, the optimizer was continuously cycling. It could not converge even when we averaged the results over three independent trainings. Consequently, we performed a simpler brute-force grid search, where the grid consisted of AB = (32, 128, 512), INT = (3, 4, 5), RB = (10, 20, 30), LR = (10^−3^, 10^−4^, 10^−5^), and BS = (2, 5, 10, 50, 100). For simplicity, we kept the remaining hyperparameters at the default values. Many of the training runs reached validation MAEs down to 0.6–2 kcal mol^−1^ for the 1k SA–W data. Subsequently, we performed the same grid search for training on 
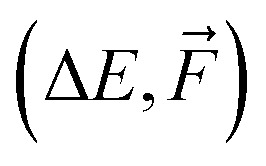
 of 1k SA–W data, on Δ*E* of 16k SA–W data, using Δ-learning on Δ*E* of 1k SA–W data, and also on Δ*E* of 1k SA–AM data. While the larger (16k) and more complex (SA–AM) dataset preferred more complex NN models, in the overall ranking, the model presented in Table 2 seems the most suitable for all the studied systems, and we will use this setup for the training of the following NNs. Although additional hyperparameter optimizations will likely not reach significantly more accurate models, they should be performed on new systems, as some of the ‘optimal’ hyperparameters are on the edge of the grid search values. The rankings are presented in the SI-2.†

**Table 2:** The final hyperparameters used for all the following models in this work. The optimized hyperparameters are highlighted in bold

Category	Hyperparameter	Value
Representation	AB = atom basis	**128**
Representation	INT = interaction layers	**5**
Representation	RB = radial basis	**20**
Representation	CUTOFF = cut off	5 Å
Model	Properties	Δ*E* or 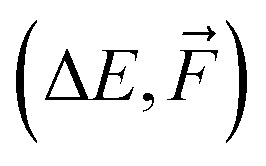
Model	Loss function	Eqn (3)
Model	Δ*E vs.* 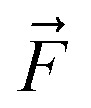 trade off *ξ*	100% or 1%
Model	Data used for validation	10%
Optimization	Optimizer	AdamW
Optimization	LR = learning rate	**0.0001**
Optimization	Learning rate scheduler	OFF
Optimization	Epochs	Varies between ∼10^2^–10^3^
Optimization	Early stopping	OFF
Optimization	BS = batch size	**2**
Initiation	RNG seed	42 or (7, 42, 69)

The final model comprises nearly 1 M trainable parameters, where this number is primarily defined by AB, INT, and RB. While we did not vary the CUTOFF, it is another crucial parameter to consider, especially for the dynamics of molecular cluster formation. Although the interaction of distant atoms within a cluster will be propagated *via* message-passing through other atoms,^71^ studying cluster evaporation or collisions using molecular dynamic simulations might be inaccurate due to the lack of long-range interactions. However, this is beyond the scope of the current manuscript. At the same time, using a large CUTOFF parameter makes the NN more complex and computationally more expensive, *i.e.*, more difficult to train. Another option for properly incorporating long-range interactions would be using a different ML model (*e.g.*, PhysNet^72^ or SpookyNet^73^) that combines short-ranged interaction modeling with small CUTOFF and long-range/dispersion corrections calculated, *e.g.*, from atomic positions and partial charges.^74,75^ Further, for minimizing the loss function, we utilize the Adam optimizer with weight decay (AdamW) and L_2_ regularization.^76^ The learning rate (LR) affects the step size of the gradient descent algorithm during training. An excessively high rate may cause overshooting or overfitting, whereas a very low rate may result in slow convergence. Although the LR of 10^−3^ has reached the best ranking in our grid search, we reduced the LR to 10^−4^ to reduce instabilities during training. Ideally, an LR scheduler should be used to progressively lower the learning rate. However, we disabled it for consistency, with the schedule multiplier set to 1. We use a very small batch size of 2 compared to the typical 100 or 200. This size represents the number of training samples used in one optimization iteration. Smaller batch sizes can lead to faster convergence as more iterations are performed within one epoch. Conversely, larger batch sizes (typically limited by computer memory) can provide more accurate iteration during optimization and, thus, can be used for fine-tuning.

#### 3.1.3 Training and validation

**Fig. 3:**
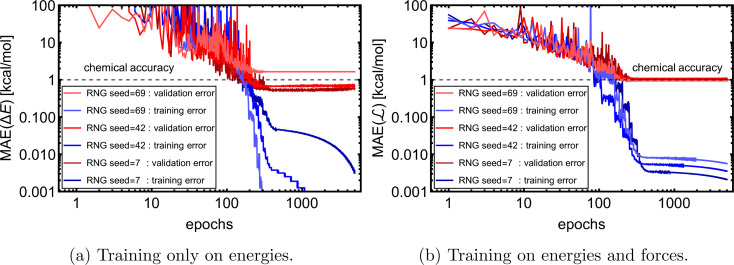
Training and validation error evolution with epochs for 1k of the SA–W system with three times differently initiated training. RNG = random number generator.

Using the optimal hyperparameters, we separately trained the NN model on Δ*E* and 
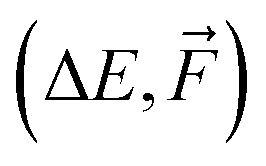
 of 1k of SA–W data. We initiated three separate training runs for each model to eliminate any randomness, using random-number-generator (RNG) seeds of 7, 42, and 69. Fig. 3 shows training and validation errors for all the runs. All models reach validation MAEs of ∼1 kcal mol^−1^. Smaller variations in validation MAEs among differently initiated models are observed when training on both energies and forces, as the loss function variation is significantly reduced by averaging over 3*N* + 1 times more values (*i.e.*, 3*N*
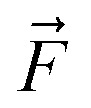
 components and 1 Δ*E*). In all cases, only ∼300 epochs are required to converge the model. After 200–300 epochs, further training appears redundant as it only overfits the training database and, in some cases, even increases the validation MAE. Due to low variations with different RNG seeds, we further only use seed 42. Note that variations among differently initiated models would become more apparent when the training database is also altered (see Section 3.4 and 3.5).

#### 3.1.4 Learning curve

To investigate the learning curve, we used the SA–W database of 18k clusters calculated at ωB97X-D/6-31++G(d,p). The same data were used in our previous work^28^ employing kernel ridge regression (KRR), implemented in the QML program.^26,27^ For consistency, we also use 520 largest SA_4_W_5_ clusters for testing and the rest for training and validation. The data sampling and simulations are repeated three times for statistics. Each model uses 1000 epochs for training. We model the electronic binding energies (Δ*E*). However, separate models are also trained on Δ*E*, 
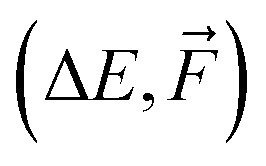
, and Δ-learning with GFN1-xTB used as the low method (see eq. (2)).

**Fig. 4:**
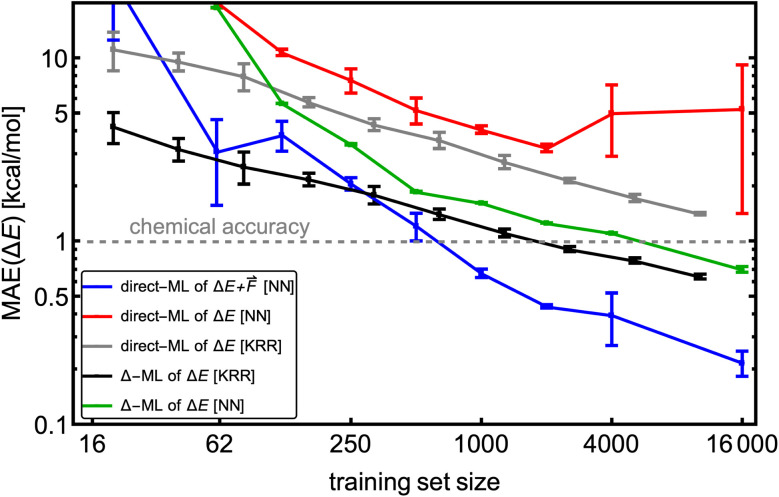
The learning curves for training on the SA–W clusters while testing on the largest SA_4_W_5_ clusters excluded from the training. Different lines correspond to direct-/Δ-learning of KRR/NN model trained on 
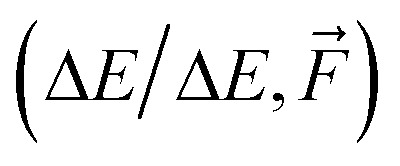
. The kernel-ridge-regression (KRR) results are taken from Kubečka *et al.*^28^ The error bars represent the standard deviation. Note the logarithmic axes.

We use the same NN model for all systems and database sizes. Fig. 4 presents all the KRR^28^ and our new NN learning curves. KRR performs better with small (<∼200) training data sizes compared to NN. For both KRR and NN, Δ-learning improves the accuracy by a factor of 2–3. When training solely on energies, this NN model achieves ∼1.2 × MAE of the corresponding KRR model with the same training set size, reaching the MAE of 0.7 kcal mol^−1^ at 16k training data. Therefore, this model reaches below the chemical accuracy of 1 kcal mol^−1^. The NN trained on both 
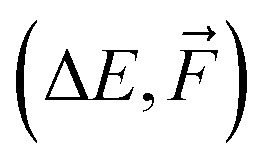
 outperforms others with MAE of Δ*E* lower than 0.3 kcal mol^−1^. Note that for NN, the mean absolute errors (MAEs) are slightly greater than the validation MAEs presented in the previous section, as the testing is performed on the largest SA_4_W_5_ clusters while validation is performed on the 10% of the data cut from the training databases.

Interestingly, direct learning of the NN model appears to be more susceptible to initialization and data choice. The large variations in the direct learning of the NN model on energies (red line) for large training sizes indicate that this choice of hyperparameters makes the model very sensitive to the initial conditions, and a search for more suitable hyperparameters at these sizes would be beneficial. Chen *et al.*^18^ also demonstrated variation in their NN modeling (with NN model termed as VSpecNN) and suggested averaging over three independent NN models reduces MAEs of energies and forces by ∼30%.

Most importantly, NN computational times for larger training sizes significantly outperform the KRR times. Also, predictions of the NN model are orders of magnitude faster than for KRR, which scales quadratically with the training set size or even cubically for large sets.

### 3.2 The model performance

**Fig. 5:**
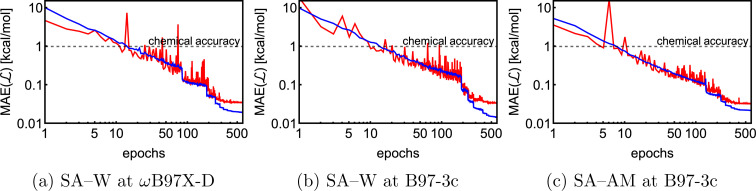
Training (blue) and validation (red) evolution of loss function for the full databases (∼18k) of the SA–W and SA–AM systems.

We trained three NN models on the full SA–W^ωB97X-D/6-31++G(d,p)^, SA–W^B97-3c^, and SA–AM^B97-3c^ databases, while training on both energies and forces. Fig. 5 shows the evolutions of the loss function, which are mainly dependent on the model and system complexity and the learning rate. Both SA–W cases behave similarly as expected due to a high correlation between the B97-3c and ωB97X-D/6-31++G(d,p) binding energies. The SA–AM system also performs similarly to the SA–W cases as the SA–AM database complexity (cluster sizes and number of atom types) appears proportionally compensated by the NN model extension with one additional atomic feature set. All training runs clearly reached a plateau after 600 epochs. Each training took nearly three days using 1 GPU and 4 CPUs. All training and validations achieved mean absolute errors below 0.1 kcal mol^−1^. The best model (*i.e.*, the model with the lowest validation loss) from each of the three training runs was subsequently used for testing.

**Fig. 6:**
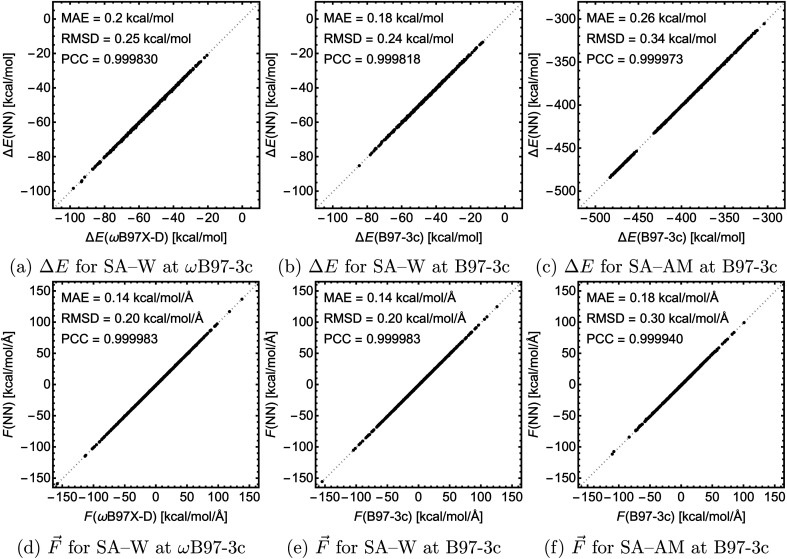
The correlation between the NN-modeled and the QC-calculated electronic binding energies and force components for the three studied cases. MAE = mean absolute error, RMSD = root mean squared difference, and PCC = Pearson correlation coefficient.

For testing, we used the largest clusters in the database which were excluded from the training, *i.e.*, SA_4_W_5_ for the SA–W systems and (SA_8_AM_10_, SA_9_AM_10_, and SA_9_AM_11_) for the SA–AM system. Fig. 6 shows the correlation of the NN-modeled and the QC-calculated energies and forces across all systems. For chemical predictions, these models are quite accurate with RMSD and MAE < 1 kcal mol^−1^, and with a very high correlation with the target method (PCC ≈ 1). The same applies to the force component predictions with RMSD and MAE < 1 kcal mol^−1^ Å^−1^ and force directions from angle deviation analysis with mean error < 1° (see SI-3†). In the case of SA–W, there is almost no apparent difference when modeling the two QC methods (ωB97X-D and B97-3c) due to their high correlation (see SI-4†). The SA–AM modeling shows only slightly larger MAE and RMSD than the SA–W modeling, likely for the same reasons as mentioned before, *i.e.*, the SA–AM database complexity appears proportionally compensated by the NN model expansion. Similar to the previous section, note that all MAEs are slightly greater than the validation MAEs (see Fig. 5) as the testing is performed on the largest clusters while validation is performed on 10% of the data cut from the training database.

### 3.3 Application to large clusters

**Fig. 7:**
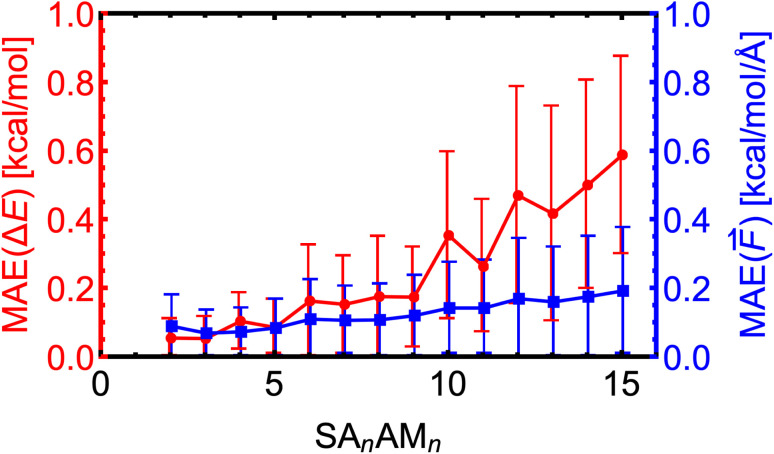
Absolute error distributions of the modeled energies and force components for the SA_*n*_AM_*n*_ clusters trained at the B97-3c level. The error bars show the standard deviation.

Here, we expand the above examination of the SA–AM model to even larger clusters and show the trend of MAEs with increasing cluster size. We took 25–50 random non-equilibrium clusters from the work of Wu *et al.*^77^ for each SA_*n*_AM_*n*_ cluster size, where *n* ∈ 2–15. Note that we only used clusters smaller than SA_10_AM_10_ for the training. In Fig. 7, we observe that the MAEs of the electronic binding energies and force components are almost linearly increasing with cluster size. This proportionality to cluster size arises from the sum of atomic contributions, with each atom likely contributing with a similar error. Additionally, the molecules are differently polarized within the large clusters, which might not be well-captured by the NN model, resulting in increasing MAE of the force components as well slightly increased growth of MAEs of the energies for the SA_10–15_AM_10–15_ clusters. Nevertheless, the MAEs consistently remain below the chemical accuracy threshold of 1 kcal mol^−1^ or 1 kcal mol^−1^ Å^−1^.

**Fig. 8:**
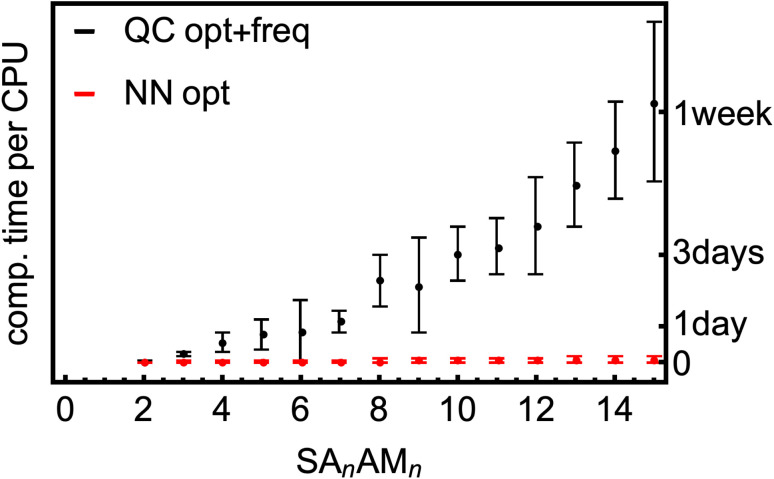
SA–AM cluster size dependence of the computational times for the optimization and vibrational frequency calculations at the B97-3c level of theory compared to the short times required for NN (pre-)optimization.

We have a fast method that quite accurately learns the reference QC method. Fig. 8 shows the typical computational times required for optimization and vibrational-frequency calculations for the SA–AM clusters during configurational sampling.^77^ Note that the times are multiplied by a factor of 8 as 8 CPUs were used even though the scaling of QC methods with the number of CPUs is not exactly linear. The frequency calculations take up approximately 15–50% of the computational time for the large clusters and up to 80% for the small. The scaling behavior of the B97-3c method is relatively moderate, exhibiting an almost linear trend in contrast to the poor scaling of other QC methods (*e.g.*, ωB97X-D or even the coupled-cluster methods). Regardless, many such calculations must be performed during thorough configurational sampling, which is computationally demanding. Utilizing the above-trained NN model for SA–AM, we also show the times required for geometry optimization with the same optimization criteria as for the QC optimization (see Fig. 8). The final geometry will be close to the true minimum, but post-optimization at the target QC level has to be applied to reach the same geometry. For example, we performed configurational sampling and took one SA_7_AM_7_ structure optimized at GFN1-xTB. Subsequent optimization of this molecule at B97-3c (within ORCA) lasted 10 CPU-hours and required ∼80 iteration steps. The RMSD of the initial and final structure is 0.28 Å. Performing optimization (within ASE) with the trained NN takes 1 min per 100 iterations and approx. 100 iterations are required to reach the same optimization criteria as in the default settings of the ORCA program. The RMSD between both final structures is 0.04 Å, and with more iterations used in the NN optimization (300), the RMSD improvement is already negligible (lowered to 0.03 Å). When taking the NN-optimized structure, less than an extra 2 CPU-hours were required for B97-3c optimization to reach the minimum structure, which differs from the fully QC-optimized structure by ∼0.01 Å in RMSD (considered as the same geometry). Although there is already a 5-fold speed-up in a single optimization, there will be a massive overall speed-up by omitting numerous energetically high-lying configurations after the NN pre-optimization. This clearly underlines that NN techniques will play an important role in future studies of large molecular clusters.

### 3.4 Molecular-cluster dynamics *via* neural network

With an NN that can model forces, we can mimic Born–Oppenheimer molecular dynamics (BOMD) simulations of clusters at a significantly lower computational cost. We used the NN model from the previous section trained on SA–AM^B97-3c^ to demonstrate this on a 100 ps long MD simulation. The simulations are performed using the Atomic Simulation Environment (ASE^78,79^), with a timestep of 0.1 fs, the Nosé–Hoover thermostat with friction frequency of 0.01 fs^−1^ and target temperatures (*T*) of 300 and 450 K. The entire 100 ps simulation only took ∼9 hours on 1 CPU. With reduced data dumping and a well-optimized simulation script, 1 ns simulation could be achieved within 1–3 CPU-days. Although the choice of thermostat or other parameters might not be ideal for real applications, this work aims to demonstrate the NN model's ability to quickly simulate large molecular clusters at the accuracy of a QC level of theory. Clearly, for MD simulations longer than a few picoseconds, the whole process of data generation, QC single-point and gradient calculation, NN training, and NN-boosted MD will become computationally faster than running BOMD at the QC level itself.

**Fig. 9:**
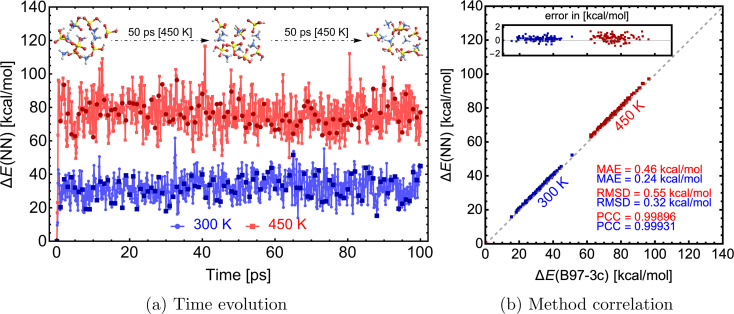
Molecular dynamics simulations of a SA_7_AM_7_ cluster with energies and forces predicted *via* the NN model. The right graph presents the correlation of NN-modeled and QC-re-calculated energies of 100 uniformly sampled structures from the MD trajectory. MAE = mean absolute error, RMSD = root mean squared difference, and PCC = Pearson correlation coefficient.

Fig. 9a illustrates the time evolution of the electronic binding energy. 100 uniformly sampled (every 1 ps step) structures were recalculated at the B97-3c level to further validate the model. Fig. 9b shows a high correlation between NN-modeled and QC-re-calculated energies. A similar satisfactory correlation is observed for the force components (see SI-5†). At the low temperature (300 K), the MAE of 0.24 kcal mol^−1^ is similar to errors observed during the comparison for structures from Wu *et al.*^77^ (see Fig. 7). With higher temperature (450 K), the model performance is decreased and the MAE of 0.46 kcal mol^−1^ is almost twice as large as the MAE at the lower temperature. This can be attributed to the difference in data generation, and higher accuracy can be gained by expanding the database with appropriate structures. In other words, the training database was constructed by extracting structures from a short MD simulation at 300 K at GFN1-xTB (see Section 2.1), but MD simulations on the B97-3c potential energy surface and even at the higher temperature of 450 K will visit untrained parts of the configurational space. When we experimented with simulations at temperatures of 500 K or more, we experienced frequent simulation failures due to molecule fragmentation caused by inaccurate NN modeling. Nevertheless, below 500 K, the molecular cluster integrity remains unchanged during these simulations.

**Fig. 10:**
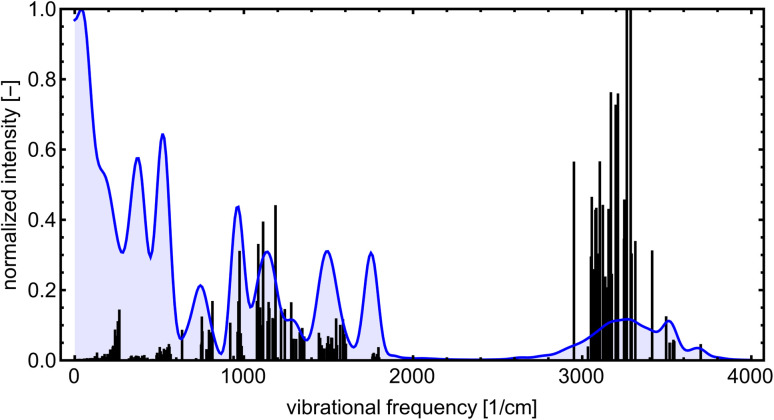
The power spectrum for SA_7_AM_7_ at 300 K obtained from ML-boosted MD simulation (blue) and spectrum of harmonic-vibrational frequencies for the lowest found energy-minimum configuration at B97-3c level (black lines). All peaks are normalized according to the highest intensity.

Even though the NN model maintains energy and force accuracy during the MD simulation, we also recommend examining whether other dynamics properties, such as radial distribution function and diffusivity, have been preserved (*e.g.*, see the work of Fu *et al.*^80^). MD simulations can enhance our understanding of many cluster properties. For instance, we used the TRAVIS program^81,82^ to analyze the vibrations stored in the MD trajectory at 300 K. Fig. 10 demonstrates the analyzed power spectrum and compares it to the equilibrium harmonic vibrational frequencies of the lowest free energy SA_7_AM_7_ conformer. The QC harmonic interpretation of the vibrational frequencies is insufficient for these weakly bound clusters, crowded with many anharmonic and low-frequency vibrations.^83^ Hence, MD simulation becomes important for interpreting some cluster behaviors. PaiNN was also designed to model directional properties such as dipole moments and polarizabilities. Modeling these properties along the generated trajectory allows calculating IR or Raman molecular spectra from molecular dynamics simulations. However, we omit them as this is beyond the scope of the current work.

### 3.5 Large database reduction

**Fig. 11:**
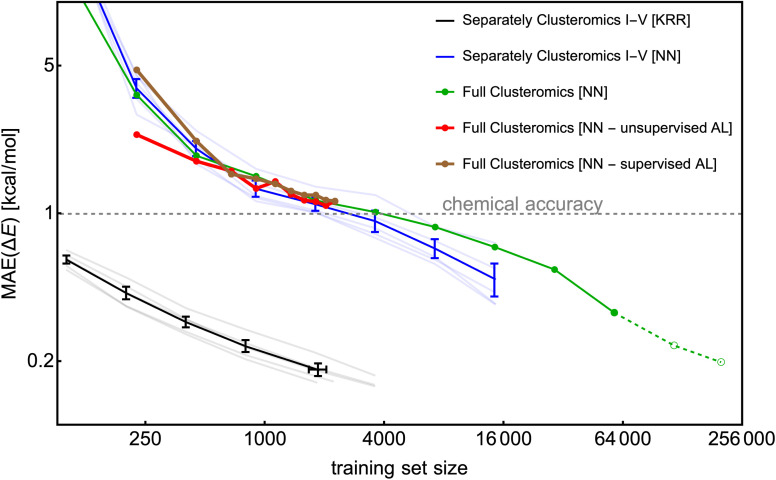
The learning curves for the KRR and NN models trained on separate/full Clusteromics I–V datasets. The error bars represent the standard deviation over the Clusteromics I–V modelings. No error bars are assigned for the full Clusteromics modeling. The last two points of NN modeling on full Clusteromics are only extrapolated estimates based on 100-epoch training.

To construct a large database, we utilize the Clusteromics I–V databases (see Fig. 1). Electronic binding energies (Δ*E*) of these data were calculated at the r^2^SCAN-3c^36^ and GFN1-xTB^50,51^ levels, making them suitable for Δ-ML. Knattrup *et al.*^32^ used 5-fold cross-validation and showed that the KRR model could reach MAEs lower than 1 kcal mol^−1^ for each Clusteromics separately when trained on very few data (∼50–100). Note that the KRR-model error is low (compared to KRR modeling in Fig. 4) as the test/validation is also performed on small clusters, *i.e.*, not only the largest clusters in the database. Knattrup *et al.*^32^ reported difficulties related to computational times for their large (>1k) training databases. Here, we performed similar training of the NN model (training for 1000 epochs) for each training set size and evaluated the model on the entire corresponding Clusteromics dataset. In Fig. 11, we demonstrate that the NN model again does not outperform the KRR model but consistently reaches the chemical accuracy of 1 kcal mol^−1^ for each Clusteromics set, with a training set requiring at least 2k of random data. We used the same ‘optimal’ hyperparameters of the NN model as in the previous sections, *i.e.*, optimized for slightly different systems, which is likely why the NN model reaches MAEs of 1 kcal mol^−1^ for 2k training dataset while the KRR model reaches 0.2 kcal mol^−1^. The KRR has a large advantage when tested on data similar to the training data. Testing on structures that are different from the training database would potentially make the KRR and NN performance more comparable for large training sets. Nevertheless, training and validation times for NN modeling again outperform KRR and are no longer the main bottleneck of ML model applications. For instance, predicting 1000 energies with the trained NN model takes a few minutes using 1 CPU, outperforming KRR by orders of magnitudes, which requires days and many CPUs for training databases with more than 1k structures.

The Clusteromics I–V database combined into one ∼221k large Clusterome database^32^ offers a playground for data filtering/reduction. Fig. 11 demonstrates our ability to train the NN model for significantly larger training sizes than the KRR model. Despite only performing one training for each training set size, we see a smooth decrease in MAE down to ∼0.3 kcal mol^−1^ for the 64k training dataset. Due to computational times, we have added the 128k and full Clusteromics MAE only based on the 100-epoch performance relative to the 64k database. Surprisingly, the MAEs are not much worse than in the case of separate Clusteromics modelings, likely due to the fact that the accuracy of electronic binding energies is mainly driven by the description of hydrogen bonds across all the Clusteromics datasets.

Finally, we applied the database reduction/active learning (AL) procedure presented by Smith *et al.*^56^ We took 0.25k data, trained the NN model, and used the predictions on the full Clusteromics database to identify the next potential candidates for training. Here, we use the terms supervised AL, where we obtain the errors by comparing the predicted Δ*E* value to the true r^2^SCAN-3c value, and unsupervised AL, where we obtain the errors as the standard deviation of the predicted Δ*E* values between three NN models (also known as ‘query by committee’), initiated with different RNG seeds. The worst performing 0.25k data are added to the training set for the next iteration, where the NN model is again fully trained from scratch. Fig. 11 shows that the two methods (red and brown lines) are sensitive to the choice of the first 0.25k data but perform quite similarly after a few iterations. Unfortunately, they do not seem to perform better than the random sampling (green line). We even tested a random sampling of 0.25k data from the worst-performing systems (*e.g.*, with error threshold >1 kcal mol^−1^) and continued training the NN model from the previous step (*i.e.*, no training from scratch) with no significant differences. We believe the database reduction will not significantly reduce MAEs for systems with similar chemical features. The driving mechanism of cluster formation is hydrogen bonding. The more training data, the more accurate the NN model. However, perhaps no particular outliers introduce hard-to-model hydrogen bonds or offer a significantly greater improvement when introduced to the training set. In SI-6,† we used KRR and tested the active learning on a small database (0.25k). There, active learning reduces the MAE by a factor of <1.5 compared to random sampling. However, active learning even reduces the maximum error by a factor of ∼2. Another option to test in the future would be selecting the worst MAEs per atom.

To conclude, although active learning appears to offer only a small MAE improvement for molecular clusters, it can help to eliminate outliers.

## 4 Conclusion

We used several databases of quantum chemistry (QC) data for typical atmospheric molecular clusters and showed that machine learning could easily substitute the computationally demanding QC calculations. Specifically, we used the polarizable atom interaction neural network (PaiNN) to model the cluster's binding energies, both with or without interatomic forces. We show that hyperparameter variation (*e.g.*, reducing batch size) leads to faster converging NN training without compromising accuracy. We demonstrate that NNs do not outperform the accuracy of the KRR modeling by Kubečka *et al.*,^28^ but the computational times for NNs are significantly lower. Similar to the case of KRR modeling, we find that Δ-learning improves the accuracy ∼4-fold. We use the energies and interatomic forces to train NN models for sulfuric acid–water clusters and sulfuric acid–ammonia clusters with quite reliable performance compared to the trained QC methods even when tested on larger structures excluded from the training: mean absolute errors of <0.26 kcal mol^−1^ for energies and <0.18 kcal mol^−1^ Å^−1^ for force components, and root mean squared displacements of <0.34 kcal mol^−1^ and ∼0.30 kcal mol^−1^ Å^−1^, respectively.

Furthermore, we show that these NN models will be very useful for application in configurational samplings of larger molecular clusters, as the atomic error contribution remains constant with increasing cluster size. While single-point energy evaluation at a high-level QC level for large clusters, with ten or more molecules, takes hours and often more than a day, the same evaluation with NN is nearly instant (∼seconds). We demonstrated this by comparing B97-3c and the NN model and showed the model's ability to optimize geometries and reproduce the B97-3c close-to-equilibrium structures.

Finally, we tested database reduction methods employing supervised and unsupervised active learning. The data reduction slightly improves the NN model performance for the same data sizes compared to random selection. We speculate that this could be caused by the fact that the driving mechanism of cluster binding is hydrogen bonding, which gets better described with more data, but there is no particular data that would introduce hard-to-model hydrogen bonds. Nevertheless, active learning appears to be a suitable tool for eliminating outliers. Furthermore, we find that the NN model trained only on energy, using Δ-learning, is able to utilize significantly more data than our previous KRR model.^32^ While we again confirm that KRR performs better for smaller sizes, NNs also can reach MAEs lower than chemical accuracy of 1 kcal mol^−1^, while outperforming KRR with respect to the computational times required for both training and predictions. We envision utilizing NN models for a better understanding of cluster dynamics. This includes cluster rigidity, reorganization after formation, reorganization before fragmentation, or even reactions within the cluster or on its surface. Overall, we believe that NN modeling will play a pivotal role in future studies of atmospheric molecular clusters.

## Data availability

All computational programs are cited within the article. Additionally, software Mathematica was used for plotting graphs and molecule visualization. All our scripts are available on GitHub (referenced in ESI†) as well as the processed data used for ML training and result data from analysis.

## Author contributions

Conceptualization: JK, ZT, JE; data curation: JK, YK; formal analysis: JK, DA, YK; funding acquisition: JK, JE, ZT; investigation: JK, DA; methodology: JK, DA, YK; project administration: JK; software: JK, ZT, DA, ME, HW; resources: JE; supervision: JE; validation: JK, DA; visualization: JK; writing – original draft: JK, JE; writing – review & editing: YK, ZT, DA, ME, HW.

## Conflicts of interest

All authors declare that they have no conflicts of interest.

## Supplementary Material

VA-003-D4VA00255E-s001
