# Supplementary material for: Accurate modeling of the potential energy surface of atmospheric molecular clusters boosted by neural networks
Source: Env Sci Adv. 2024 Aug 13;3(10):1438–51. doi: 10.1039/d4va00255e (PMC11334116; doi:10.1039/d4va00255e)
Supplement: VA-003-D4VA00255E-s001 [file VA-003-D4VA00255E-s001.pdf]

Supplementary Information (SI) for Environmental Science: Advances.
This journal is © The Royal Society of Chemistry 2024

Supporting Information for Accurate Modeling of the Potential Energy Surface of Atmospheric Molecular Clusters Boosted by Neural Networks

Jakub Kubečka,*,† Daniel Ayoubi,† Zeyuan Tang,‡ Yosef Knattrup,† Morten Engsvang,† Haide Wu,† and Jonas Elm†

†*Department of Chemistry, Aarhus University, Langelandsgade 140, 8000 Aarhus C, Denmark*

‡*Center for Interstellar Catalysis, Department of Physics and Astronomy, Aarhus University, Ny Munkegade 120, 8000 Aarhus C, Denmark*

E-mail: ja-kub-ecka@chem.au.dk

Phone: +420 724946622

SI-1 Package and Data Availability

The JKML package comes together with JKCS and is a free, open-source program immediately available at

https://github.com/kubeckaj/JKCS2.1

The package contains Bash and Python codes and is suitable for any GNU/Linux system. Each script contains its own help function, and the overall manual with some recommended approaches is available at

https://jkcs.readthedocs.io

The Atmospheric Cluster Database 2.0 is available at

https://github.com/elmjonas/ACDB.git

The models and the full databases can be found under the Articles/kubecka24_neural_network folder.

SI-2 Rankings of Hyperparameter Optimization Searches

We have performer optimization of batch size (BS), learning rate (LR), atomic (feature) basis (AB), number of interaction NN layers (INT), and radial basis (RB) hyperparameters. The performance was quantified by comparing the loss function (LOSS) after 200 training epochs. The hyperparameters that ranked high for all tested systems were chosen for all the NN models within the main text. The methods' rankings are also in the ACDB database under the Articles/kubecka24_neural_network folder and are located in the Additional_files/Hyperparemeter_Optimization subfolder.

SI-3 Force vector angle deviation analysis

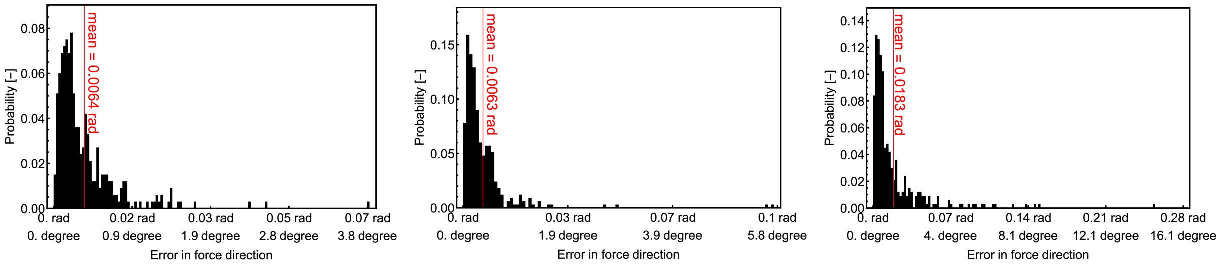

(a) SA–W at ωB97X-D (b) SA–W at B97-3c (c) SA–AM at B97-3c

Figure SI-1: Distribution of errors and mean error of the NN-predicted force vector direction as an angle difference from the true direction.

SI-4 ωB97X-D/6-31++G(d,p) and B97-3c Correlation

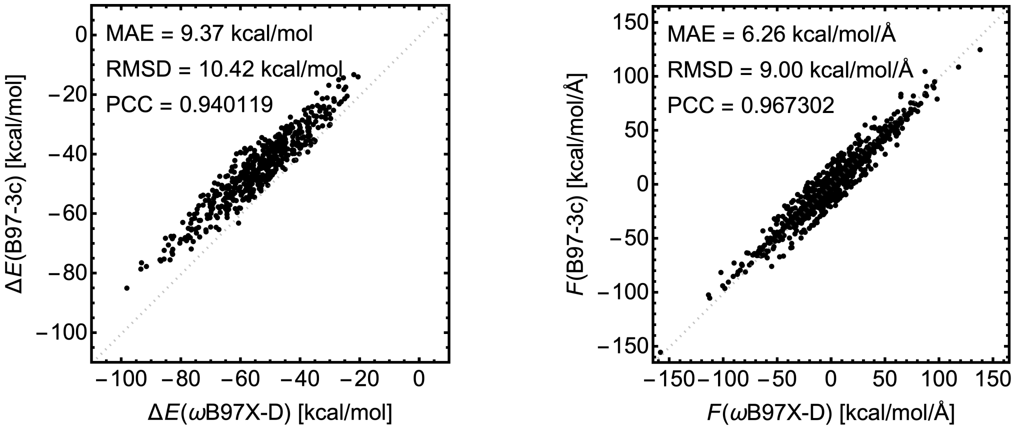

(a) Energy correlation (b) Force component correlation

Figure SI-2: Correlation between ωB97X-D/6-31++G(d,p) and B97-3c electronic binding energies and force components for the SA–W system. MAE = mean absolute error, RMSD = root mean squared difference, and PCC = Pearson correlation coefficient.

SI-5 Performance of Force Modeling

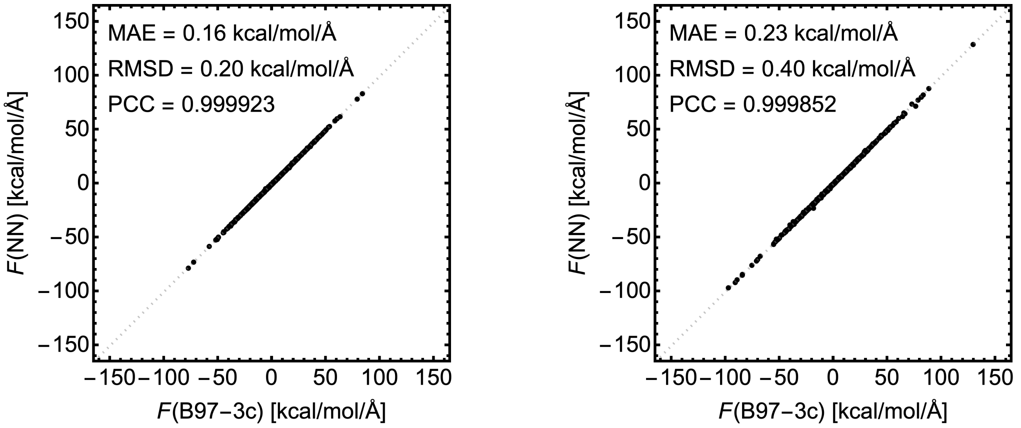

(a) Correlation for simulation at 300 K. (b) Correlation for simulation at 450 K.

Figure SI-3: Correlation between the NN-modeled and QC-calculated (B97-3c) force components for 100 structures uniformly sample from the NN-boosted MD simulation of the SA_7AM_7 cluster. Only 1000 random force components are visualized. MAE = mean absolute error, RMSD = root mean squared difference, and PCC = Pearson correlation coefficient.

SI-6 Data Reduction Test

We tested several methods of database reduction. For simplicity and to save computational power, we only performed a database reduction of 0.25k database, which was randomly pre-sampled from the Clusteromics database. Also, only one test was performed (i.e., no statistics). The kernel ridge regression (KRR) method was used as it performs well even for small training set sizes. The database reduction methods we tested are:

- random sampling (yellow line)
- uniform sampling based rescaled mass and energy properties (black line)
- uniform sampling based rescaled radius of gyration and energy properties (green line)
- uniform sampling based rescaled radius of gyration and energy properties while the monomers were always forced to be in the database (orange line)
- selection subset of the smallest clusters in the dataset (excluding monomers) (brown line)

- selection subset of the largest clusters in the dataset (purple line)
- active learning, where 10 first structures are sampled randomly, and the training dataset for the next iterations is always enlarged by 10 worst performing structures (excluding the training ones) from the ML energy predictions within the previous step (yellow line)
- active learning, where 10 first structures are sampled randomly, and the training dataset for the next iterations is always enlarged by 10 randomly sampled structures from bad performing (errors >1 kcal/mol) structures (excluding the training ones) from the ML energy predictions within the previous step (pink line)

Figure SI-4 shows the mean absolute errors (MAEs) and standard error deviations (SEDs) dependence on the training set size. Clearly, active learning outperforms other database reduction methods. However, the improvement is not that marginal. Note that in order to get a 0.1k training database for the active learning, we had to perform 9 other trainings in a series beforehand. Except for this disadvantage, active learning could be used to reduce the maximal errors in the training database, which we present via a steep decrease in standard error deviation.

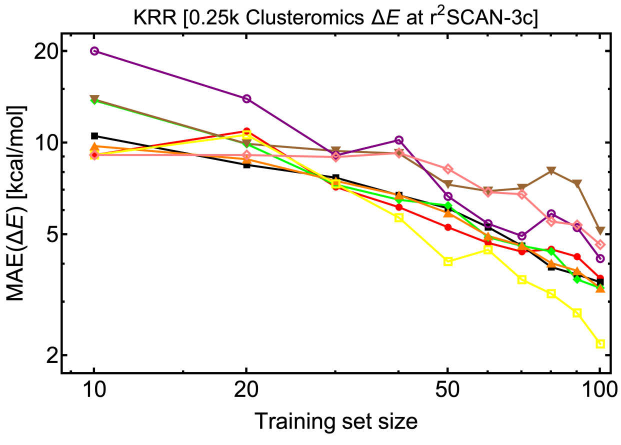

(a) Mean absolute errors.

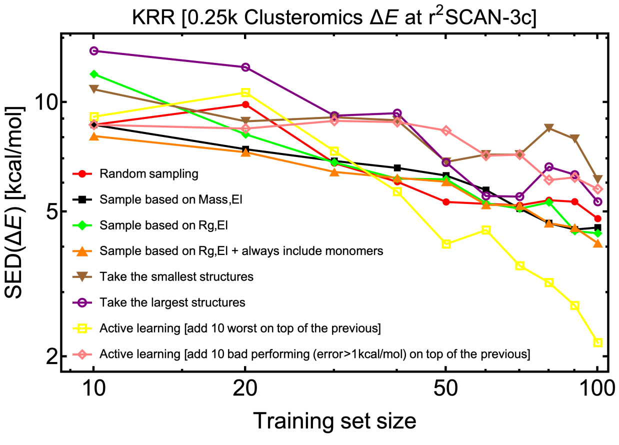

(b) Standard error deviation.

Figure SI-4: Performance of different data reduction methods from 0.25k randomly pre-sampled (same 0.25k used in all tested cases) Clusteromics structures. Here, KRR is used as the ML method, the test is always performed on the full 0.25k data, and only the binding energy (ΔE) at r^{2}SCAN-3c is modeled.
